# Effects of Hydrostatic Pressure on Growth and Luminescence of a Moderately-Piezophilic Luminous Bacteria *Photobacterium phosphoreum* ANT-2200

**DOI:** 10.1371/journal.pone.0066580

**Published:** 2013-06-20

**Authors:** Séverine Martini, Badr Al Ali, Marc Garel, David Nerini, Vincent Grossi, Muriel Pacton, Laurence Casalot, Philippe Cuny, Christian Tamburini

**Affiliations:** 1 Aix Marseille Université, CNRS/INSU, IRD, Mediterranean Institute of Oceanography (MIO), UM 110, Marseille, France; 2 Université du Sud Toulon-Var, CNRS/INSU, IRD, Mediterranean Institute of Oceanography (MIO), UM 110, La Garde, France; 3 Laboratoire de Géologie de Lyon, UMR5276 Université Lyon1, CNRS, Villeurbanne, France; 4 ETH Zürich, Geological Institute, Zürich, Switzerland; Loyola University Medical Center, United States of America

## Abstract

Bacterial bioluminescence is commonly found in the deep sea and depends on environmental conditions. *Photobacterium phosphoreum* ANT-2200 has been isolated from the NW Mediterranean Sea at 2200-m depth (*in situ* temperature of 13°C) close to the ANTARES neutrino telescope. The effects of hydrostatic pressure on its growth and luminescence have been investigated under controlled laboratory conditions, using a specifically developed high-pressure bioluminescence system. The growth rate and the maximum population density of the strain were determined at different temperatures (from 4 to 37°C) and pressures (from 0.1 to 40 MPa), using the logistic model to define these two growth parameters. Indeed, using the growth rate only, no optimal temperature and pressure could be determined. However, when both growth rate and maximum population density were jointly taken into account, a cross coefficient was calculated. By this way, the optimum growth conditions for *P. phosphoreum* ANT-2200 were found to be 30°C and, 10 MPa defining this strain as mesophile and moderately piezophile. Moreover, the ratio of unsaturated vs. saturated cellular fatty acids was found higher at 22 MPa, in agreement with previously described piezophile strains. *P. phosphoreum* ANT-2200 also appeared to respond to high pressure by forming cell aggregates. Its maximum population density was 1.2 times higher, with a similar growth rate, than at 0.1 MPa. Strain ANT-2200 grown at 22 MPa produced 3 times more bioluminescence. The proposed approach, mimicking, as close as possible, the *in situ* conditions, could help studying deep-sea bacterial bioluminescence and validating hypotheses concerning its role into the carbon cycle in the deep ocean.

## Introduction

The deep sea, under 1000-m depth, is characterized by a high hydrostatic pressure (≥10 MPa), with, generally, a low temperature and a low organic-matter concentration. Laboratory experiments using pure cultures of piezophilic bacteria have highlighted microbial adaptations to high hydrostatic pressure. The adaptive traits include those related to growth [1], [2], membrane [3] and storage lipids [4], membrane and soluble proteins [5], [6], the respiratory-chain complexes [7], [8], replication, transcription and translation [9], [10]. Most isolated piezophilic bacteria belong to the genera: *Carnobacterium, Desulfovibrio, Marinitoga*, *Shewanella, Photobacterium, Colwellia, Moritella*, and *Psychromonas* within the Gammaproteobacteria subclass reviewed by Bartlett *et al.* [11].

Darkness is another major characteristic of this deep-sea environment that can be disturbed by a biological phenomenon named bioluminescence. Bioluminescence is the process by which living micro- or macro-organisms emit light. Amongst the bioluminescent organisms, marine luminous bacteria are ecologically versatile and can be found as free-living forms, epiphytes, saprophytes, parasites, symbionts in the light organs of fishes and squids, and commensals in the gut of various marine organisms [12], [13], [14]. Metagenomic analysis from deep eastern-Mediterranean water samples shows a surprising high number of *lux* genes directly involved in bioluminescence [15]. As far as we know, all-known marine bioluminescent bacteria are phylogenetically affiliated to the *Vibrio*, *Photobacterium* and *Shewanella* genera within the Gammaproteobacteria subclass [16]. Amongst them, *Photobacterium phosphoreum* is the predominant species found in the Mediterranean Sea [17].

Those of the most studied micro-organisms are, for piezophily, *Photobacterium profundum* SS9 (e.g. [18]), not known as luminous, and for bioluminescence, *P. phosphoreum* (e.g. [19]). Up to date, little information is available concerning potential physiological-adaptation mechanisms of luminous bacteria to hydrostatic pressure, especially for both piezophily and bioluminescence. In this study, we used a bioluminescent strain isolated from Mediterranean deep-sea waters (sampled at 2200-m depth) and identified as *Photobacterium phosphoreum* ANT-2200 [20]. At this depth, the *in situ* conditions of pressure and temperature are about 22 MPa and 13°C, respectively. The purpose of this study is (1) to define temperature and pressure optima for growth and (2) to study pressure effect (0.1 versus 22 MPa, 13°C) on growth and bioluminescence activities of *P. phosphoreum* ANT-2200 using a new laboratory controlled hyperbaric system dedicated to high-pressure and bioluminescence studies.

## Materials and Methods

### Growth parameters of *P. phosphoreum* ANT-2200 under various temperature and hydrostatic-pressure conditions

*P. phosphoreum* ANT-2200 (GenBank accession number EU881910) was isolated from sea water collected in the Northwestern Mediterranean Sea at the ANTARES neutrino-telescope site (42°54′N/06°06′E) at 2200-m depth (13°C) (see [20] for details). Phenotypic and enzymatic characterizations are available in Supporting Information (Table S1). Procedures for pre-culturing were performed as described by Al Ali *et al.* [20]. For the determination of growth rate and maximum population density as a function of pressure and temperature, mid-log cultures were inoculated 1∶10 into 5-mL sterilized syringes supplied with 3/4 of seawater complete medium (SCW medium) and with 1/4 oxygen-saturated Fluorinert™ FC-72 (3 M™). The impoverished SCW liquid medium contained per liter (pH 7.5): 3 mg of yeast extract, 5 mg of bio-peptone, 3 mL of glycerol, 250 mL of distilled water, and 750 mL of old sea water [21]. Fluorinert™ FC-72 was used as oxygen supplier to ensure the growth and the luminescence of the bacterial strain in closed conditions [22], [23], [24].

Triplicates cultures were incubated at pressures of 0.1, 10, 22, 30 and 40 MPa and for temperatures of 4, 13, 20, 30 and 37°C. Syringes were placed into high-pressure bottles (HPBs). In order to avoid decompression-recompression of the samples, each HPB corresponded to one incubation time. Bacterial growth was estimated by measuring the optical density (OD_600 nm_) using a spectrophotometer (Perkin Elmer, Lamda EZ201 UV/Vis spectrophotometer).

Direct counting was also performed using epifluorescence microscopy with DAPI-staining procedure, according to Porter and Feig [25]. This counting method was used to define the link between total-cell counts (DAPI counts) and optical density (OD_600 nm_) according to the equation (1). For DAPI-cell counts, to avoid possible artefact due to the aggregates, the samples were sonicated (3 min), vortexed (1 min), diluted with milliQ-water, then, sonicated (2 min), vortexed (1 min) and finally filtered on 0.2- µm-pore-size polycarbonate filters. The data have been, firstly, treated separately for atmospheric-pressure (0.1 MPa) and high-pressure (22 MPa) conditions. Since no significant difference has been observed between the two sets of data, a common relation has been defined as following:
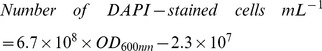
(1)

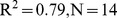



Traditionally, a linear regression is used to determine the growth rate of a strain during the logarithmic phase. The logistic (or Verhulst) model [26] was used in this study to determine both the growth rate (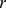) and the maximum population density (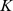). This model gives a continuous function of optical density, fitting discrete experimental data measured during the bacterial growth. Its hypotheses take into account limited resources in the medium and are defined as:

The birth rate: 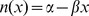



The mortality rate: 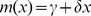


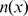
and
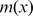
are linear functions with 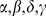
four real numbers and 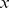
is the population density. The birth and mortality rates are supposed to be constant during time:



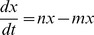



Meaning that the logistic model is written as:
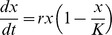
Where:
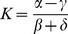
K, being the maximum population density (expressed in optical density, OD_600 nm_) that *P. phosphoreum* ANT-2200 can reach according to the growth conditions (temperature, pressure.) and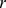, being the growth rate, defined as:
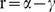



Biologically, the intrinsic growth rate (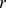, expressed in h^−1^) is supposed to be positive (meaning that α>γ).

A cross coefficient (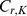) has been calculated for both temperature and pressure effects on growth. If *R* and *K* are two 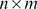 matrices with *n* the number of temperature and *m* the number of pressure conditions tested, the C_r,K_ is defined as: 
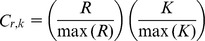
With 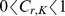


The values for growth rate (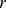, h^−1^), maximum population density (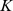, OD_600 nm_) and cross coefficient (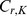) were used to construct extrapolated-contour plots for the pressure-temperature dependency using R software [27].

### Scanning electron and transmission electron microscopes

Cultures of *P. phosphoreum* ANT-2200 were performed at 0.1 and 22 MPa at 13°C. Cells were harvested at the end of the logarithmic phase and prepared for electron microscopy in order to observe cellular morphology and structure according to the pressure conditions.

Scanning Electron Microscopy (SEM) was performed according to two different procedures. On the one hand, cells were fixed with 0.2% glutaraldehyde, filtered on 0.2- µm-pore-size nucleopore membranes, washed with filtered and sterilized seawater with 2% osmic acid, and then with MilliQ water. Washed cells were dehydrated and observed using SEM (FEI Quanta 250 FEG, Centre Technologique des Microstructures, University Claude-Bernard, Lyon 1). On the other hand, cells were rapidly frozen in liquid nitrogen and lyophilized for 48 h using a CHRIST beta 2–4 LT+LD lyophilizator, operated at a temperature of −50°C and a pressure of 4 Pa. After complete dehydration, samples were attached onto stubs with double-sided adhesive (carbon type) and sputter coated, in a Baltec MED020 Sputter Coater, with a thin film of platinum to improve electrical conductivity of the sample surface. Samples were subsequently observed using SEM (FEI Quanta 250 FEG, Centre Technologique des Microstructures, University Claude-Bernard, Lyon 1).

Transmission Electron Microscopy (TEM) was carried out using cells fixed with 2% glutaraldehyde, buffered with PBS and embedded in 2% agar. Cells were post-fixed in 1% osmium tetroxide, dehydrated in a graded series of ethanol and embedded in Epon. Sections of 70 nm were realized using an ultramicrotome (Leica ultracut S), contrasted with uranyl acetate and lead citrate, and observed under a Philips CM 120 Transmission Electron Microscope at 80 kV.

### Cellular fatty-acid composition of *P. phosphoreum* ANT-2200 grown at 0.1 and 22 MPa (13°C)

Cultures of *P. phosphoreum* ANT-2200 were grown in 300-ml completely-filled polyethylene bottles (188 mL of culture +62 mL of oxygenated Fluorinert™ FC-72), at 13°C and at 0.1 or 22 MPa (into high-pressure bottles). The bottle stoppers were equipped with a septum through which the pressure was applied. Cells in the late logarithmic stage of growth were harvested by centrifugation (20 min, 5500 rpm at 0°C). Bacterial pellets were immediately frozen at −20°C and lyophilized. Lipids were extracted using the modified method of Bligh and Dyer [28] with dichloromethane/methanol/water (DCM/MeOH/H_2_O, 1∶2∶0.8, v/v/v) under sonication. Following the addition of DCM and water to allow phase separation (final DCM/MeOH/H_2_O ratio of 1/1/0.9), the lower DCM layer was collected and the upper aqueous phase was further extracted with DCM (x2). The combined lipid extracts were concentrated, dried over anhydrous sulfate and evaporated to dryness (N_2_ flux) before being trans-esterified (50°C, 2 h) with 2% sulfuric acid in MeOH in the presence of toluene [29]. Individual fatty acids were identified and quantified by gas-chromatography-mass spectrometry (GC-MS), using an Agilent 6890 N gas chromatograph interfaced to an Agilent 5975 mass spectrometer (electronic impact at 70 eV). The GC was equipped with a splitless injector and a HP5-MS capillary column (30 m×0.25 mm×0.25 µm). Helium was used as the carrier gas (constant flow of 1 mL min^−1^) and the oven temperature was programmed from 70 to 130°C at 20°C min^−1^, and then at 4°C min^−1^ from 130 to 300°C at which it was hold for 20 min.

### Bioluminescence of *P. phosphoreum* ANT-2200 at 0.1 and 22 MPa (13°C)

**Figure 1:**
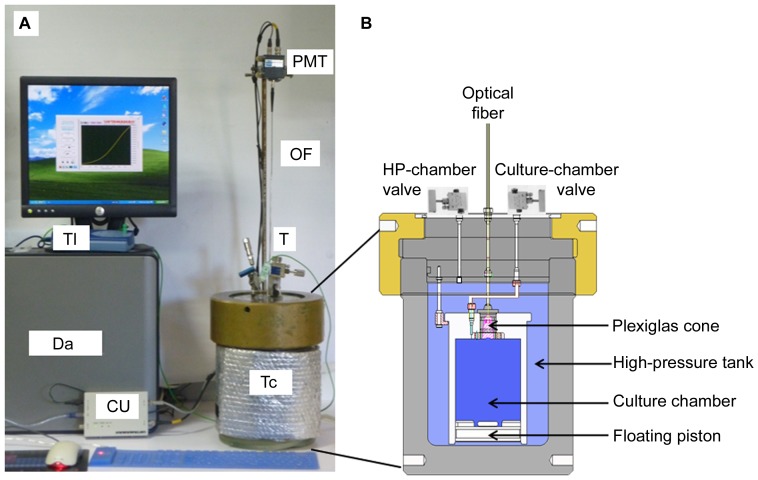
High-pressure bioluminescence system. (A) Photography of the high-pressure bioluminescence system and (B) schematic section diagram of the high-pressure bioluminescence tank. PMT: photomultiplier tube; OF: optical fiber; CU: photomultiplier counting unit; T: high-pressure temperature sensor; Tc: Tubing around tank for temperature control connected to a thermo chiller (not shown); Tl: Data logger for temperature sensor; Da: PC for data acquisition of bioluminescence and temperature;

Bioluminescence (photons sec^−1^) was monitored with a high-pressure bioluminescence system shown in Figure 1 A. Luminous bacteria were cultivated within a culture chamber placed inside a high-pressure tank (Fig. 1 B). The hydrostatic pressure is transmitted (via the HP-chamber valve) from the high-pressure tank to the culture chamber via a floating piston (Fig. 1 B). Sub-sampling is done by opening the culture-chamber valve, while the pressure is monitored, using a piloted pressure generator [30] connected to the HP-chamber valve. The culture chamber is made in ertalyte (chemically and biologically inert material, white for light reflection) and sustains a plexiglass cone which transmits photons emitted by luminous bacteria by the way of an optical fiber (Fig. 1 B). Photon counting was obtained by integrating signals during 10 seconds using a photomultiplier (H7155, Hammamatsu®) linked to its counting unit (C8855, Hammamatsu®). Temperature was regulated using an external housing of tubing around the high-pressure bioluminescence tank. Temperature was controlled with a thermo chiller and monitored using a K-type thermocouple directly fitted within the high-pressure tank. More details of the high-pressure bioluminescence tank can be found in [20]. Experiments were performed three times for 0.1 and 22 MPa.

## Results and Discussion

### Growth temperature and pressure optima of *P. phosphoreum* ANT-2200

**Figure 2:**
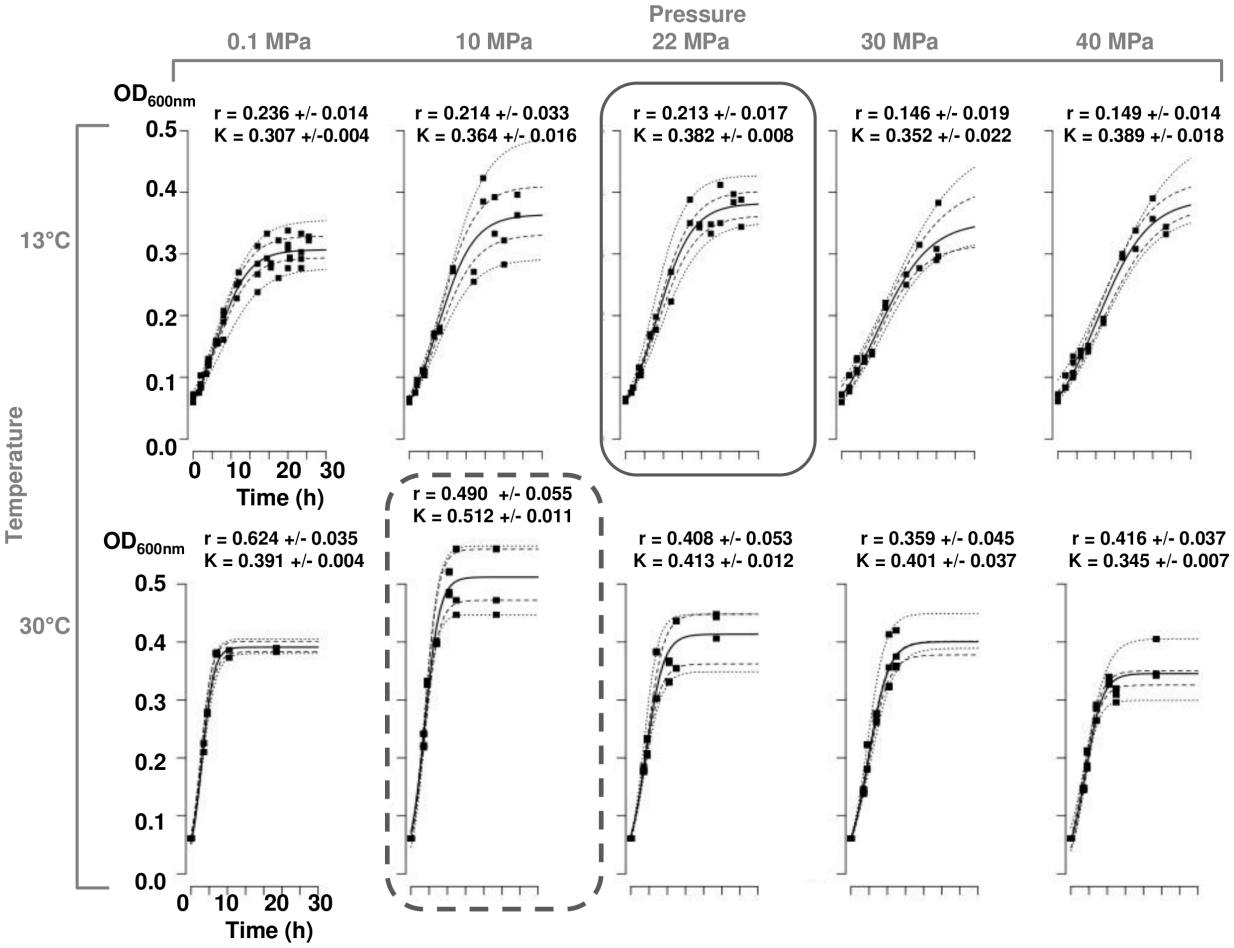
Example of logistic model fitting empirical growth data of *P. phosphoreum* ANT-2200. Experiments were done at pressures of 0.1, 10, 22, 30 and 40 MPa and at temperatures of 13°C and 30°C. The logistic model (line) improves the r and K parameter estimation on empirical growth data (dots). Dashed lines are levels of confidence for the 0.05 and 0.95 quantile curves and the 0.25 and 0.75 quantile curves. Mean +/− standard deviation for growth rate (r, h^−1^) and maximum population density (K, OD_600 nm_) parameters are indicated. The dotted frame is the growth curve under optimum pressure and temperature conditions using both r and K parameters. The solid line frame is the growth curve under *in situ* conditions, at 22 MPa and 13°C. N is the number of replicates done for the same pressure and temperature conditions.

Using the logistic model, growth-rate (r expressed as h^−1^) and maximum-population-density (K expressed as OD_600 nm_) parameters were defined for each temperature (4, 13, 20, 30, 37°C) and pressure (0.1, 10, 22, 30, 40 MPa) conditions (Fig. S1). Figure 2 presents the model curves fitting with empirical data obtained at 13°C and 30°C, for all tested pressures. The model parameters have been estimated qualitatively using the confidence interval of the logistic growth curves.

**Figure 3:**
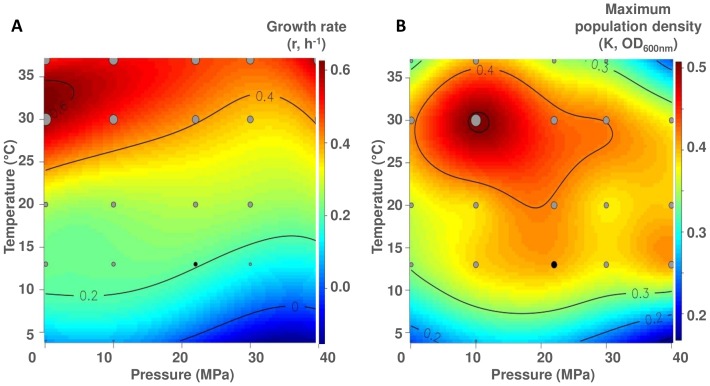
Extrapolated-contour diagram of the temperature-pressure dependence of *P. phosphoreum* ANT-2200. The diagrams are plotted for (A) the growth rate (r, h-1) and (B) the maximum population density (K, OD600 nm). The grey circles indicate parameter values used to extrapolate the contours. Size is proportional to their value. The black circle corresponds to the *in situ* conditions for the strain. Isolines define zones with same level of parameter values.

**Figure 4:**
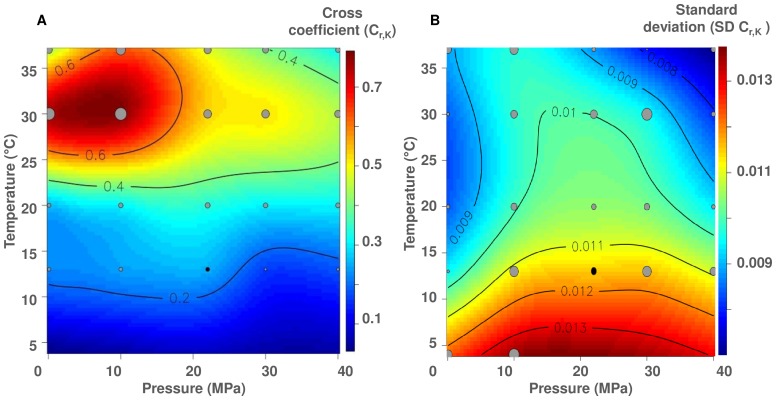
Cross diagram of the temperature-pressure dependence of *P. phosphoreum* ANT-2200. (A) Extrapolated-contour diagram of the temperature-pressure dependence for both the growth rate (r, h^−1^) and the maximum population density (K, OD_600 nm_) for *P. phosphoreum* ANT-2200. The cross coefficient C_r–K_ is defined as: 0<C_r–K_<1. (B) Standard deviation associated to the C_r–K_ coefficient. The grey circles indicate parameter values used to extrapolate the contours. Size is proportional to their values. The black circle corresponds to the *in situ* conditions for the strain. Isolines define zones with same level of values.

The r and K parameters were used to construct the extrapolated-contour diagram of their temperature-pressure dependence (Fig. 3 A and Fig. 3 B, respectively). *P. phosphoreum* ANT-2200 was able to grow at hydrostatic pressures ranging from 0.1 to 40 MPa and at temperatures ranging from 4 to 37°C. The strain grew very slowly at 4°C, with a minimum r value obtained at 0.1 MPa (0.058 ± 0.014 h^−1^) and a low K of 0.167 ± 0.032 (Fig. 3 A). The higher r values were observed at 30°C/0.1 MPa and at 37°C/40 MPa (0.624 ± 0.035 h^−1^ and 0.596 ± 0.038 h^−1^ respectively, Fig. 3 A). While growth rates appeared to depend on temperature, pressure did not clearly affect it, at least in the tested range (Fig. 3 A). Furthermore, the stationary phase was very short with fast and strong cell lyses at 37°C for all pressures (Fig. S1). In opposition to what is generally observed [3], [31], it was not possible to define the pressure affinity of strain ANT-2200 using the growth rate only. Therefore, we also used the same approach to overlook the effect of temperature and pressure conditions on maximum population density reached by strain ANT-2200. Interestingly, both influenced K and for all tested conditions, the highest value (0.512 ± 0.011 OD_600 nm_) was observed at 30°C and 10 MPa (Fig. 3 B). While the growth rate is commonly the main growth parameter used in microbiology, our experiments show that the maximum population density has a strong influence on the definition of the optimal conditions for growth. So, we propose to cross the r and K parameters (using the C_r–K_ coefficient) in order to define these optima (in our case, temperature and pressure). An extrapolated-contour diagram was drawn for the cross coefficient C_r–K_ (Fig. 4 A). The standard deviation, associated to the C_r–K_ coefficient, was calculated using the confidence-interval estimation on parameters from the logistic model and illustrated in Figure 4 B. The standard-deviation values were one order below the cross-coefficient values, meaning that the cross-coefficient interpretation was robust. The highest C_r–K_ coefficient value (0.78) was found at 30°C and 10 MPa (Fig. 4 A). These optima allowed characterizing strain ANT-2200 as mesophile and moderately piezophile [32]. As previously observed [3], the optimal pressure for piezomesophiles is often found lower than their habitat pressure, while their optimal temperature is higher than their habitat temperature. Different hypotheses can be evoked to explain the temperature shift [33] (1) inheritance from ancestors who lived in a warmer environment or (2) life in warmer temperatures in the gut of deep-sea animals. Even if we identified optimal conditions for growth at 30°C and 10 MPa, we decided to perform further experiments at *in situ* conditions (13°C) and to compare atmospheric pressure (0.1 MPa) to high pressure (22 MPa). This allowed to checking the piezophilic character of strain ANT-2200 and studying its morphology and its fatty-acid composition, known to be affected by hydrostatic pressure [10], [34], [35]. The pressure-dependent (0.1 versus 22 MPa, 13°C) bioluminescence activity of *P. phosphoreum* ANT-2200 was also characterized.

### Morphology of *P. phosphoreum* ANT-2200

**Figure 5:**
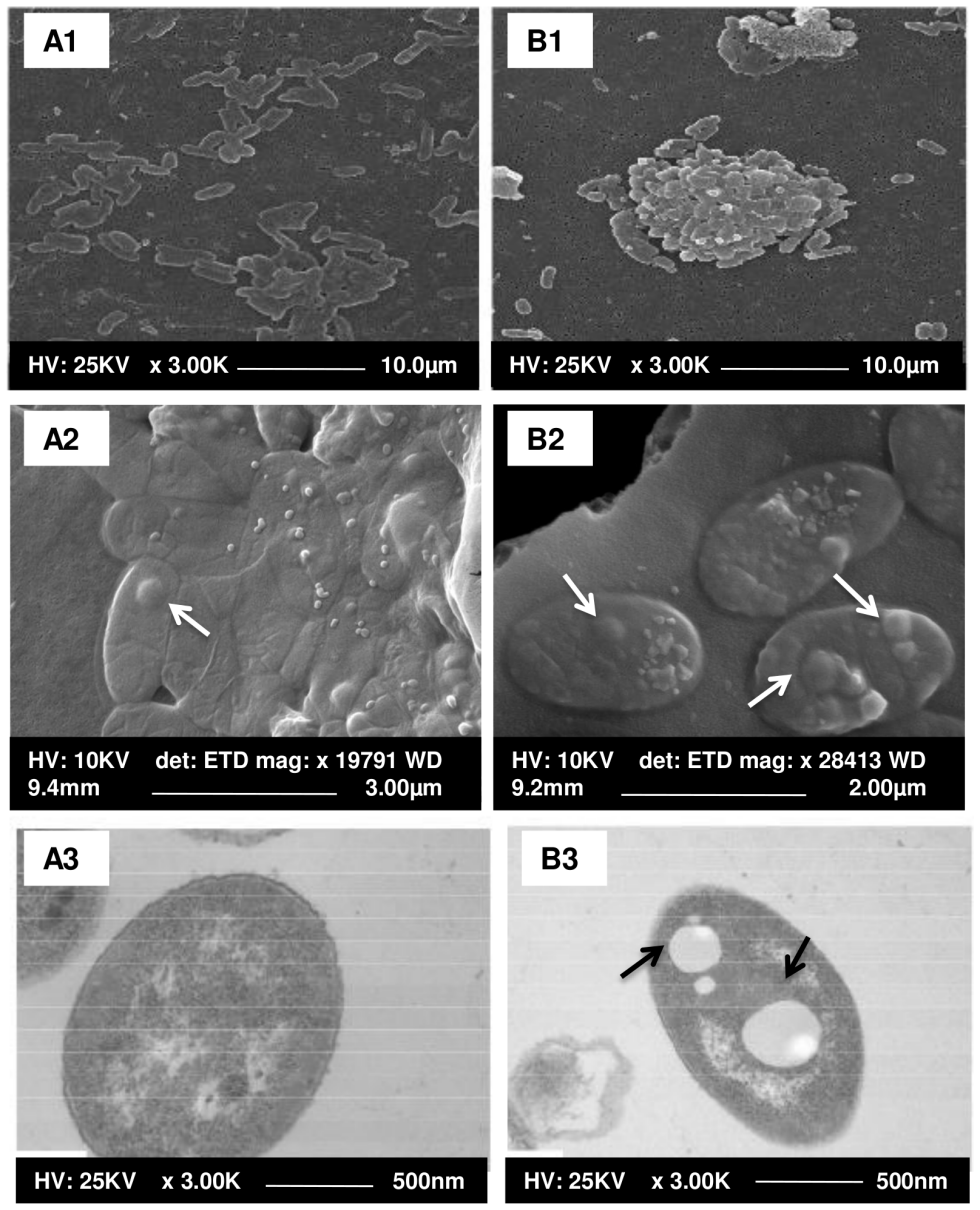
Micro-photographs of *P. phosphoreum* ANT-2200 cells by electron microscopy. Observation at 0.1 MPa (A1 on dehydrated samples, A2 on freeze-dried samples) and 22 MPa (B1 on dehydrated samples, B2 on freeze-dried samples) using SEM and at 0.1 MPa (A3) and 22 MPa (B3) using TEM. Intracellular inclusions are indicated by arrows.

*P. phosphoreum* ANT-2200 was observed by SEM and TEM after cultivation at 0.1 and 22 MPa (Fig. 5). *P. phosphoreum* ANT-2200 is a rod-shaped bacterium. The size of the cells is in average equal to 2.4±1.4×10^−1^ µm long and 0.8 ± 0.7×10^−1^ µm wide at atmospheric pressure (Fig. 5 A1-3). Cells at 22 MPa display a smaller size (i.e., less than 2 µm long) and contain numerous intracellular inclusions (Fig. 5 B1-3). The exact nature of these inclusions has not yet been determined. Such inclusions may serve as energy reserve, may contribute directly to the metabolic capabilities of the cell, and/or may be involved in the cell ability to cope with changing environmental conditions [36], [37]. In any case, this confirms an adaptation strategy of *P. phosphoreum* ANT-2200 cells to high hydrostatic pressure. Besides, cells also appear to aggregate more at 22 MPa than at 0.1 MPa (Fig. 5 A1-B1).

### Pressure effects on the cellular fatty-acid composition of *P. phosphoreum* ANT-2200

**Figure 6:**
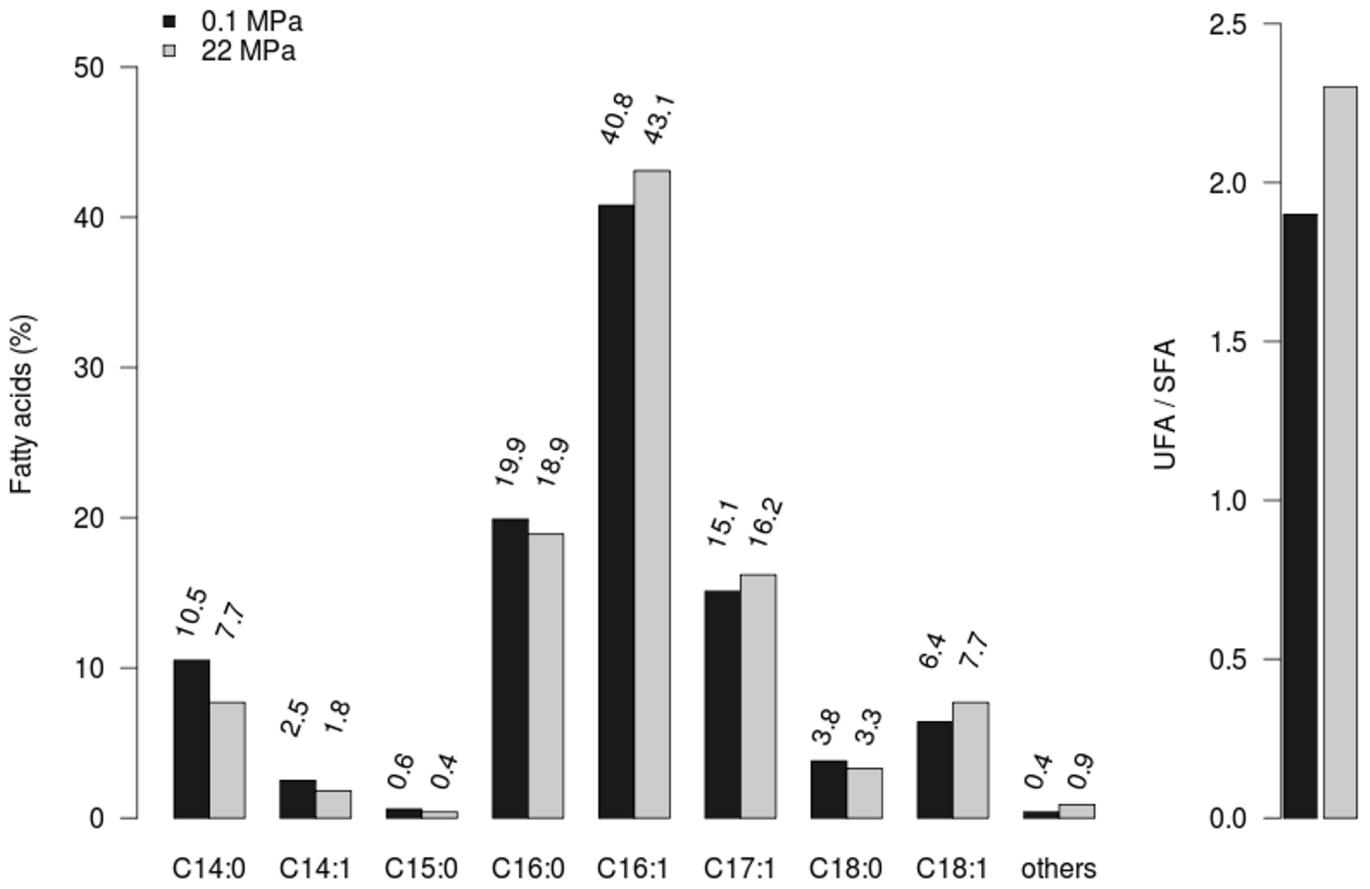
Relative total fatty-acid composition (%) of *P. phosphoreum* ANT-2200. Strain ANT-2200 is grown at 0.1 (black bar) and 22 MPa (grey bar). Others: sum of C17:0, C18:2 and C 19:1 fatty acids; UFA: unsaturated fatty acids; SFA: saturated fatty acids.

The effect of hydrostatic pressure on the cellular fatty-acid composition of *P. phosphoreum* strain ANT-2200 was determined for cultures grown at 0.1 and 22 MPa at 13°C (Fig. 6). The main fatty acid was C16:1, representing 40.8 and 43.1% of the total cellular fatty-acid at 0.1 MPa and 22 MPa, respectively. Growth at 22 MPa also induced an increase in the relative proportions of C16:1, C17:0, C17:1, C18:1 and C18:2 fatty acids. The ratio of total unsaturated vs. total saturated fatty acids (UFA/SFA) was 1.9 at 0.1 MPa and 2.3 at 22 MPa. These values are similar to those found by DeLong and Yayanos [38] for the piezophilic bacterium CNPT-3 grown under similar pressures. The increase in the relative proportions of mono-unsaturated fatty acids at elevated pressure is in good agreement with previous studies [39], [40], indicating that many piezophilic bacteria respond to an increase in hydrostatic pressure by modifying their membrane lipid composition. This homeoviscous adaptation allows to tailor the membrane to environmental conditions with suited physical properties [35]. The results obtained with *P. phosphoreum* ANT-2200 further argue for the piezophilic character of this bioluminescent strain. It is noteworthy that the poly-unsaturated C20:5 fatty acid (C20:5 PUFA or eicosapentaenoic acid) was not detected in this strain. DeLong *et al.* [41] suggested that C20:5 PUFA could be used to define strains originating from low temperature and high pressure environments. Nevertheless, the absence of such PUFA in *P.phosphoreum* ANT-2200 may be due to its origin from warmer deep-sea waters (Mediterranean Sea, average temperature about 13°C), and to its optimal temperature of growth (30°C).

### Pressure effects on bioluminescence of *P. phosphoreum* ANT-2200

**Figure 7:**
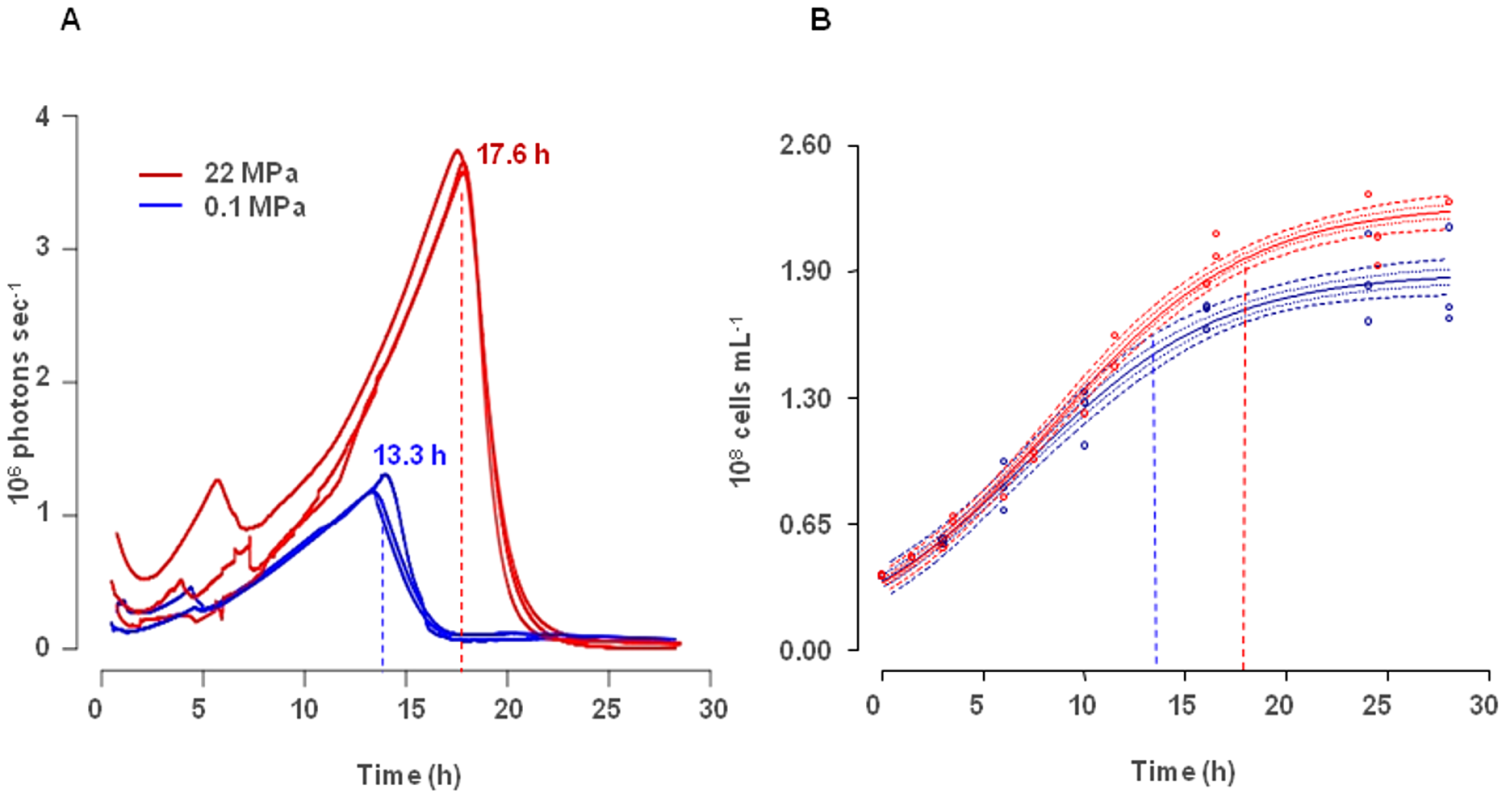
Bioluminescence and growth of *P. phosphoreum* ANT-2200. (A) Bioluminescence (photons sec^−1^) of *P. phosphoreum* ANT-2200 at 0.1 MPa (blue lines) and 22 MPa (red lines). (B) Fitted logistic growth curves for 0.1-MPa experiments (blue lines) and 22-MPa experiments (red lines). The dashed lines represent levels of confidence for the 0.05, 0.95 and 0.25, 0.75 quantile curves. Cell number is estimated using equation (1). On (A) and (B) blue and red dotted lines represent the mean time of the bioluminescence peak for both pressure conditions.

Three successive experiments using the high-pressure bioluminescent tank were carried out in order to quantify the luminescence produced by *P. phosphoreum* ANT-2200, at 0.1 MPa and 22 MPa, 13°C. Our results showed that the higher maximum cell density at 22 MPa than at 0.1 MPa is associated to higher luminescence intensity. Maximum luminescence intensity is reached, in average, at 17.6 h at 22 MPa and at 13.3 h at 0.1 MPa (Fig. 7 A). The average value of the maximum luminescence for the three replicates is three times higher at 22 MPa (3.5±0.1×10^6^ photons sec^−1^) than at 0.1 MPa (1.2 ± 0.2×10^6^ photons sec^−1^). When bioluminescence is maximal, the total cell number is 1.40 ± 0.01×10^8^ cells mL^−1^ at 0.1 MPa and 1.90±0.01×10^8^ cells mL^−1^ at 22 MPa (Fig. 7 B). At this time, the light emission capacity represents 8.4×10^−3^ photons cell^−1^ mL^−1^ at 0.1 MPa and 19.0×0^−3^ photons cell^−1^ mL^−1^ at 22 MPa. Noticeably, the ratio of photons emitted per cell and volume unit is higher at 22 MPa than at 0.1 MPa, clearly indicating the pressure dependence of bioluminescence. Since strain ANT-2200 is characterized as piezophile, its light emission appeared to be an adaptive trait more than a stress response to pressure as suggested by Czyz *et al.* [42].

Actually, during the growth, the respiration and the bioluminescence emission are two processes competing for the consumption of oxygen [43], [44], [45], [46]. To explain the differences in light emission between high pressure and atmospheric conditions, the oxygen availability has been checked. An oxygen optode (PreSens® GmbH) permitted to control the remaining presence of enough oxygen at the end of the growth (oxic condition) both under atmospheric and high-pressure conditions (data not shown). The oxygen concentration seems not to explain the differences in bioluminescence emission per cell described in these experiments. A second explanation to these results is based on the ecological aim of the bioluminescence emission. Metabolic processes, such as the increase of bioluminescent-bacterium biomass, will increase the luminescence by an autoinduction phenomenon. Many bacteria use this cell-density-dependent signalling system, also called quorum sensing, to coordinate the expression of the genes involved in biofilm formation and luminescence production [47]. In our study, the aggregates formed at 22 MPa (Fig. 5 B1) keep the cells close together, miming a higher cell density, and this could possibly induce a quorum-sensing response leading to higher bioluminescence intensity. This is in agreement with previous hypotheses from Pooley [48].

Three different ecological niches with high cell density, enhancing quorum sensing and indirectly bioluminescence, have been described in the literature so far. Firstly, light organs of marine squids or fish contain up to 10^11^ cells mL^−1^ of luminescent bacteria. This symbiosis provides an advantage for the host (prey or partner attraction.) and an ideal growth environment for bacteria [49], [50]. Secondly, marine snows are millimetre- to centimetre-size aggregates of macroscopic flocculent particles consisting of detritus, inorganic particles and phytoplankton on which micro-organisms grow [51], [52], [53]. Bacteria can develop swimming behavior to colonize this sinking organic material, therefore reaching a cell density 100 to 10,000 times higher than in the water column (up to 10^8^ to 10^9^ cells mL^−1^) [54], [55]. At this density, they are able to emit light in order to attract preys. Then, they might be ingested by macro-organisms to live in a better-growing environment [56], [57]. Thirdly, luminous bacteria are known to be present in the gastro-intestinal tracts of marine organisms. Their expelled faecal pellets are enriched in micro-organisms, including bioluminescent bacteria, up to 10^5^ to 10^6^ times more than the surrounding waters [56], [57], [58]. Ingestion rate and cycling of pellet constituents are affected by bioluminescence phenomenon [58], suggesting that bioluminescence bacteria might play an important role in the carbon cycle in the deep ocean.

### Conclusion

The strain *P. phosphoreum* ANT-2200 was isolated from a deep-water sample (2200 m, 13°C, 22 MPa) close to the ANTARES site in the Mediterranean Sea. It has been shown that, using only growth rate, it was not possible to characterize the strain growth optima. However, using both growth rate and maximum population density of strain ANT-2200, optimal temperature and pressure have been estimated at 30°C and 10 MPa. As observed in other deep-sea strains, the ratio of total unsaturated vs. total saturated fatty acids is higher at elevated pressure. All these points converge to characterize this strain as mesophile and moderately piezophile. The strain ANT-2200 produces higher luminescence intensity at high pressure (22 MPa) than at atmospheric pressure (0.1 MPa). To our knowledge, this is the first time that such phenomenon is described. Genetic determinism and corresponding ecological benefit of this pressure-controlled bioluminescence still have to be determined.

## Supporting Information

Figure S1 **Representation of growth curves for temperatures of 4, 13, 20, 30 and 37°C and for pressure of 0.1, 10, 22, 30, and 40 MPa.** The logistic model (line) improves the r and K parameter estimation on empirical growth data (dots). Dashed lines are levels of confidence for the 0.05 and 0.95 quantile curves and the 0.25 and 0.75 quantile curves. The growth rate (r, h^−1^) and maximum population density (K, OD_600 nm_) parameters are indicated.
(TIFF)

Table S1 **Phenotypic and enzymatic characterizations of** ***P. phosphoreum*** **strain ANT-2200.** The presence of cytochrome oxidase was tested with one- or two-days cultures on SWC medium, using filter papers dropped with 1% N, N-dimethyl-p-phenylene-diamine hydrochloride (Kovacs oxidase test). Catalase activity was determined by looking at bubble production in a 3% (v/v) hydrogen-peroxide solution. Physiological and biochemical characterizations of *P. phosphoreum* ANT-2200 were performed using the API 20 NE, Biolog GN2 microplates, and APIZYM methods (DSMZ, Germany).
(DOCX)
