# Supplementary figures and images for: Effects of Hydrostatic Pressure on Growth and Luminescence of a Moderately-Piezophilic Luminous Bacteria Photobacterium phosphoreum ANT-2200
Source: PLoS One. 2013 Jun 20;8(6):e66580. doi: 10.1371/journal.pone.0066580 (PMC3688590; doi:10.1371/journal.pone.0066580)

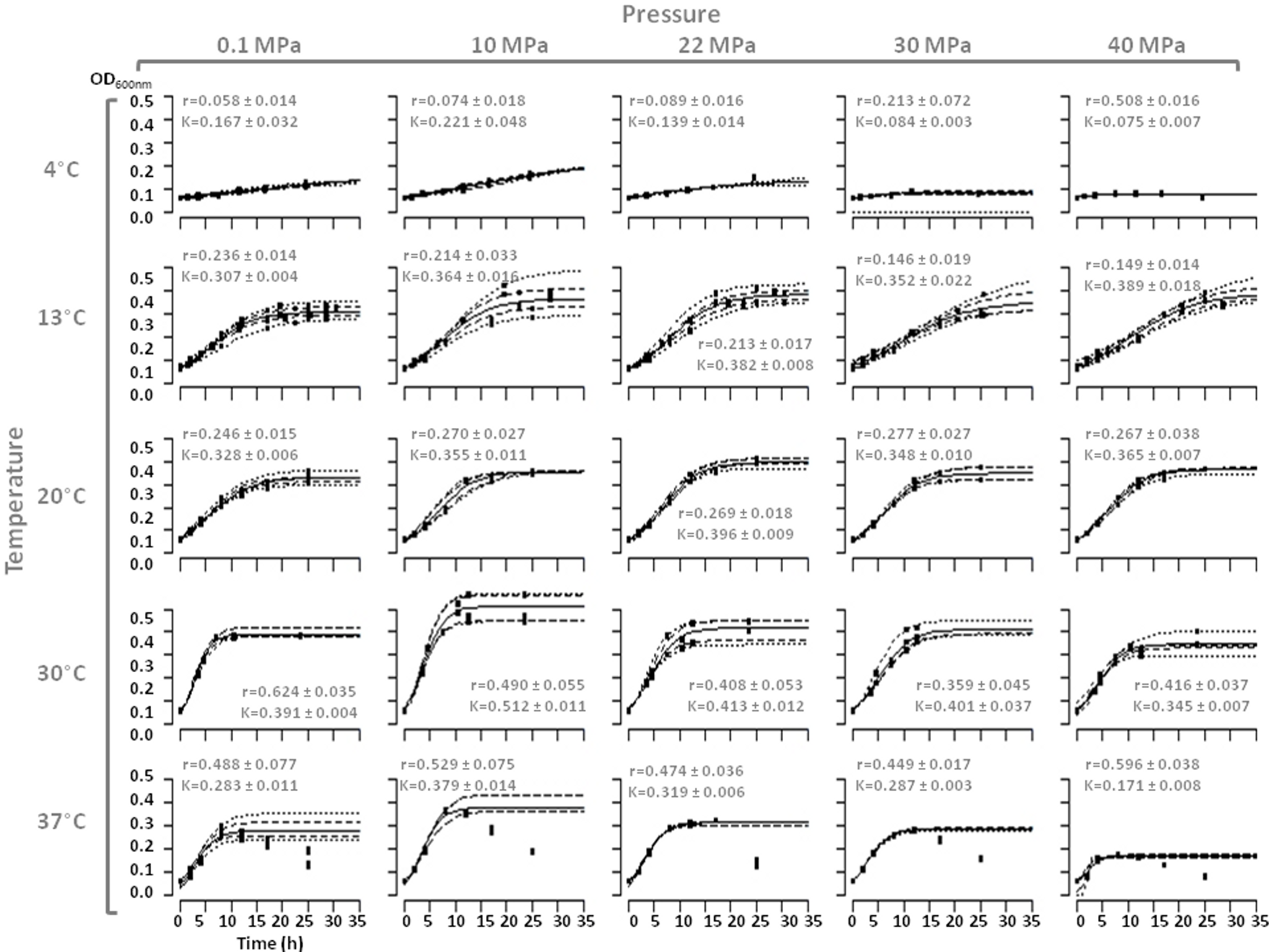

Supplement: Figure S1 — Representation of growth curves for temperatures of 4, 13, 20, 30 and 37°C and for pressure of 0.1, 10, 22, 30, and 40 MPa. The logistic model (line) improves the r and K parameter estimation on empirical growth data (dots). Dashed lines are levels of confidence for the 0.05 and 0.95 quantile curves and the 0.25 and 0.75 quantile curves. The growth rate (r, h−1) and maximum population density (K, OD600 nm) parameters are indicated. (TIFF) [file pone.0066580.s001.tiff]
