# Supplementary material for: Effects of Hydrostatic Pressure on Growth and Luminescence of a Moderately-Piezophilic Luminous Bacteria Photobacterium phosphoreum ANT-2200
Source: PLoS One. 2013 Jun 20;8(6):e66580. doi: 10.1371/journal.pone.0066580 (PMC3688590; doi:10.1371/journal.pone.0066580)
Supplement: Table S1 — Phenotypic and enzymatic characterizations of P. phosphoreum strain ANT-2200. The presence of cytochrome oxidase was tested with one- or two-days cultures on SWC medium, using filter papers dropped with 1% N, N-dimethyl-p-phenylene-diamine hydrochloride (Kovacs oxidase test). Catalase activity was determined by looking at bubble production in a 3% (v/v) hydrogen-peroxide solution. Physiological and biochemical characterizations of P. phosphoreum ANT-2200 were performed using the API 20 NE, Biolog GN2 microplates, and APIZYM methods (DSMZ, Germany). (DOCX) [file pone.0066580.s002.docx]

|  | ***P. angustum* ATCC 25915^1^** | ***P. damselae* ATCC 33539^1^** | ***P. histaminum* JCM 8968^1^** | ***P. leiognathi* ATCC 25521^1^** | ***P. profundum* SS9^1^** | ***P. profundum* DSJ4^1^** | ***P. phosphoreum* ATCC 11040^1^** | ***P. phosphoreum* 04104^2^** | ***P. phosphoreum* ANT-2200^3^** |
| --- | --- | --- | --- | --- | --- | --- | --- | --- | --- |
| N-acetyl-D-galactosamine | + | + | + | + | - | - | - | - | - |
| N-acetyl-D-glucosamine | + | + | + | + | + | - | + | + | + |
| L-arabinose | - | - | - | - | - | - | - | - | - |
| Cellobiose | - | + | + | - | + | - | - | - | - |
| D-fructose | + | + | + | + | + | - | + | + | + |
| D-galactose | + | + | + | + | + | + | - | + | + |
| α-D-glucose | + | + | + | + | + | + | + | + | + |
| α-D-lactose | - | - | - | - | - | - | - | + | - |
| Maltose | + | + | + | + | + | + | + | + | - |
| D-mannose | + | + | + | + | + | + | + | + | + |
| D-raffinose | - | - | - | - | - | - | - | - | - |
| L-rhamnose | - | - | - | - | - | - | - | - | - |
| Sucrose | + | - | - | - | - | - | - | - | - |
| D-trehalose | - | - | + | - | + | + | - | + | + |
| Turanose | + | + | + | + | - | - | - | - | - |
| Adonitol | - | - | - | - | - | - | - | ND | - |
| Myo-inositol | - | - | - | - | + | - | - | ND | - |
| D-mannitol | - | - | - | - | + | + | - | + | - |
| D-sorbitol | - | - | - | - | - | - | - | - | - |
| Glycerol | + | + | + | + | + | + | - | + | + |
| Glycogen | + | + | + | + | + | + | + | + | - |
| Tween 40 | - | + | - | - | + | + | + | + | - |
| Tween 80 | - | - | - | - | + | + | - | + | - |
| Indole production | - | - | - | - | + | + | - | ND | - |
| Glucose fermentation | - | + | - | + | + | W+ | + | + | + |
| Catalase | - | + | + | - | + | + | + | ND | + |
| Oxidase | - | + | + | + | + | + | + | + | + |
| Nitrate reduction | - | + | + | + | + | + | + | ND | + |
| Arginine dihydrolase | + | + | + | + | + | + | + | ND | + |
|  |  |  |  |  |  |  |  |  |  |

^1^ Nogi Y, Masui N, Kato C (1998) *Photobacterium profundum* sp. nov., a new, moderately barophilic bacterial species isolated from a deep-sea sediment. Extremophiles 2: 1-7.

^2^ Deng H, He C, Zhou Z, Liu C, Tan K, et al. (2009) Isolation and pathogenicity of pathogens from skin ulceration disease and viscera ejection syndrome of the sea cucumber *Apostichopus japonicus*. Aquaculture 287: 18-27.

^3^ This study

+ Positive

- Negative

W weakly

ND not determined
